# Advances in Natural Building and Construction Materials

**DOI:** 10.3390/ma18245651

**Published:** 2025-12-16

**Authors:** Paweł Strzałkowski, Luís Sousa, Ekin Köken

**Affiliations:** 1 Department of Mining, Faculty of Geoengineering, Mining and Geology, Wroclaw University of Science and Technology, Wybrzeże Wyspiańskiego 27, 50-370 Wrocław, Poland; 2 Department of Geology and Pole of CGeo—Geosciences Center, University of Trás-os-Montes e Alto Douro, 5000-801 Vila Real, Portugal; lsousa@utad.pt; 3 Materials Science and Nanotechnology Engineering Department, Abdullah Gül University, Kayseri 38100, Turkey; ekin.koken@agu.edu.tr

In the contemporary context, there is increasing pressure to reduce the environmental impacts of civil engineering while maintaining the durability and safety of constructed buildings. In this context, the appropriate use of construction and natural building materials is vital to implementing the concept of sustainable development [1,2,3,4,5]. These materials form the basis of many traditional and modern solutions in architecture, civil and hydraulic engineering, and their physical and mechanical properties make them essential elements in the pursuit of more environmentally friendly construction technologies [6]. Conversely, the necessity of utilising these materials in the refurbishment of structures, particularly those of historical and cultural significance, positions them as a pivotal element within the domain of building and construction materials [7,8,9]. The dynamic evolution of the industry, coupled with the rising demand for mineral raw materials, highlights the need for rapid and precise evaluations on the quality of aggregates and natural stones. This requirement arises from the reality that the performance and long-term behaviour of these materials depend not only on the inherent physical and mechanical characteristics but also on the specific conditions of their intended uses [10,11,12,13,14,15,16,17,18].

In the face of these challenges, contemporary research focuses on a comprehensive analysis of natural building materials, assessing their properties and forecasting the economic, environmental and social aspects of their use. Concerning the improvement of research methods, instruments and computer-aided technologies, the monitoring and conservation of ancient temples, historical structures and architectural sites necessitate pursuing solutions that harmonise technical imperatives with environmental responsibility and maintain cultural heritage.

The main objectives of this Special Issue are twofold. First, it aims to place the above-mentioned topics within a systematic framework of modern and sustainable engineering disciplines. Second, it seeks to highlight the importance of using natural materials in construction and architecture by indicating potential future research directions and their applications.

In this context, this Special Issue includes 12 articles (11 research and 1 review) [19,20,21,22,23,24,25,26,27,28,29,30], all directly related to the field of natural building and construction materials from various perspectives. The research focuses on assessing the technical properties of these materials and developing new or improved building and construction products. The main findings and achievements of this Special Issue are outlined below.

Correa-Jaramillo and Hernández-Olivares [19] investigated the development of geopolymer coating bricks incorporating ceramic waste as a sustainable alternative to conventional construction materials. The study evaluates the physicochemical characteristics of ceramic residues and their suitability for geopolymerisation. XRD and XRF analyses confirm that the waste material contains sufficient quartz (SiO_2_) and alumina (Al_2_O_3_) to form high-strength aluminosilicate structures. The resulting bricks meet the required compressive strength, although additional optimisation is necessary to achieve the desired flexural strength. Thermal performance tests and energy simulations indicate that the bricks are suitable for architectural envelope applications. Efflorescence does not affect mechanical performance and can be mitigated through hydrophobic treatments, supporting the feasibility of these geopolymerised bricks as an environmentally responsible construction solution.

Łucarz et al. [20] focused on the recovery of high-purity quartz sand from spent moulding sand using combined thermal and mechanical treatments. When organic binders are present, regeneration is relatively straightforward: with appropriate temperatures, the organic components are effectively burnt off, and any combustion residues are removed through abrasion in the fluidised-bed process. However, when the binder contains both organic and inorganic components, regeneration becomes more challenging. Although thermal treatment eliminates the organic fractions, inorganic residues remain adhered to the grain surfaces and are extremely difficult to remove. Despite these limitations, advances in material-processing and recycling technologies offer promising prospects. As natural quartz sand resources become increasingly scarce, spent moulding sands—particularly those stored in landfills—may become valuable secondary raw materials suitable for a range of industrial applications.

Ferraz et al. [21] examined the feasibility of converting land and sea snail shell waste into valuable lime-based products. Through calcination, the shells are transformed into quicklime, which is subsequently used to prepare milk of lime and hydrated lime. The chemical composition, thermal decomposition characteristics, purity, and reactivity of lime products derived from different shell types were evaluated. The results indicate that snail-shell-based lime meets or surpasses standard specifications for industrial applications, with good reactivity and whiteness. The research demonstrates a practical valorisation pathway for shell waste, reducing environmental burdens associated with aquaculture and food-processing residues. This contributes to circular economy strategies by replacing mineral-derived limestone with renewable biogenic sources.

Berger et al. [22] investigated the potential use of two types of filter dust generated during calcined clay production as supplementary cementitious materials (SCMs). The study demonstrates that one of the dusts exhibits sufficient pozzolanic reactivity to be used directly as an SCM, with performance comparable to that of conventional fly ash. The materials were classified into two categories: a reactive pozzolanic material and a predominantly inert filler with limited pozzolanic activity. Consequently, the authors provided valuable insights into the physical properties of both untreated and heat-treated oven dusts. For both material groups, increasing calcination temperature resulted in a reduction in specific surface area and water demand. The study further discusses the influence of calcination temperature on particle-size distribution and highlights opportunities to enhance reactivity through thermal optimisation. Overall, the findings indicate that certain filter dusts can be effectively valorised as SCMs, contributing to more sustainable cement production.

Dal Poggetto et al. [23] conducted a systematic study on the dissolution of two volcanic ashes in concentrated alkali. Dissolution in NaOH and sodium silicate solution is the first step in the geopolymerisation process, which, after hardening at room temperature, results in solid, durable building blocks. Generally, dissolution in a strong alkaline environment appeared unaffected by NaOH concentration (provided it was above 8 M) or by powder size (provided it remained below 75 µm) but was influenced by time. The hardened alkali-activated materials exhibit good reticulation, as indicated by the low weight loss in water (10 wt.%) upon hardening at 25 °C. The same advantage was observed in the mechanical performance of room-temperature consolidated specimens in terms of resistance to compression (4–6 MPa). Therefore, studying the alkaline dissolution of volcanic ash offers a valuable way to predict and optimise the reactivity of its phases, especially the amorphous ones.

Wang and Xue [24] studied the potential of red mud and steel slag (RM/SS) to produce a geopolymer suitable for solidifying and stabilising Pb-contaminated soil. The authors reported that, at a liquid–solid ratio of 0.76, an RM content of 79.82%, and an alkali activator modulus of 1.21, the solidified soil achieves a maximum uniaxial compressive strength (UCS) of 3.42 MPa and a Pb immobilisation efficiency of 71.95%. The resulting geopolymer matrix is primarily composed of calcium aluminosilicate hydrate, calcium silicate hydrate, and nekoite. Lead retention occurs mainly through lattice incorporation, where geopolymerisation forms Al–O–Pb and Si–O–Pb bonds. Additional Pb is immobilised through physical encapsulation within the matrix and by precipitation as Pb(OH)_2_ under highly alkaline conditions.

Gomah et al. [25] explored potential changes in mineralogy, texture, physical, and mechanical properties of Egyptian granodiorites under high temperatures up to 800 °C. Their detailed laboratory test results showed that the decrease in water content and differences in thermal stress between rock-forming minerals led to the formation of initial intergranular and transgranular microcracks in granodiorites at 200 °C and 400 °C, respectively. On the other hand, the UCS of granodiorites appears to increase up to 400 °C due to thermal hardening and the closure of initial and induced intergranular microcracks. After that temperature, the UCS decreases dramatically under the influence of unstable crack growth.

Zagaroli et al. [26] reported the mechanical characteristics of a mixed cement–lime mortar by varying the volume fractions of air lime, cement, and sand. Their laboratory test results demonstrated that using different mixtures in cement–lime mortars could offer several advantages in modern masonry construction units.

Valido et al. [27] investigated the impacts of salt crystallisation on the physico-mechanical properties of two ignimbrites from the Canary Islands (Spain). Although the mechanical properties of UCS and flexural strength (FS) decreased slightly, the rock property most affected by the salt ageing tests was the open porosity.

Brzyski and Boris [28] conducted a parametric study on potential enhancements of selected mortar properties. They used hydrated lime, metakaolin, and casein, an organic polymer derived from cow’s milk, as variable input parameters. They found that the strength properties of mortars decreased as casein content increased. Additionally, no significant relationship was observed between casein content and the drying-shrinkage behaviour of mortars.

Based on extensive previous experience with natural stone applications, Strzałkowski et al. [29] proposed a series of quantitative classifications to assess the quality of natural stones. These classifications are directly connected to fundamental physical (apparent density, open porosity, water absorption), mechanical (UCS, FS), abrasion (Böhme abrasion value), and ageing (freeze/thaw resistance) tests. The researchers also discussed enhancing the proposed classification systems by applying them across a wide range of geological settings, including igneous, metamorphic, and sedimentary rocks.

Finally, Coviello et al. [30] reviewed the use of polyethylene terephthalate (PET) in reinforcing cementitious mixtures. Based on a comprehensive literature review on the potential impacts of PET in various forms on the hardening process (density, water absorption, and shrinkage), which directly relate to the mechanical properties such as UCS and FS of concrete mixtures, the researchers concluded that, due to the hydrophobic nature of PET, the air voids in concrete mixtures tend to increase with increasing the PET content. The density of concrete mixtures can be reduced or optimised by considering different PET additives. Based on this finding, lighter construction materials can be developed through a detailed analysis of PET from various perspectives.

The results and key achievements reported in the papers published in this Special Issue highlight the significance of natural materials, with particular emphasis on sustainable construction, the proper use of natural stones, and parametric experimental studies aimed at developing novel or improved geomaterials. These efforts not only support the preservation and conservation of historical monuments and buildings but also identify the practical implications of using natural resources through rigorously designed laboratory investigations. The published studies demonstrate that using natural and laboratory-produced geomaterials can significantly reduce the environmental impacts of the construction sector while maintaining, or even improving, the functional and aesthetic performance of structures. Moreover, the contributions offer valuable insights into material characterisation, durability, and long-term behaviour assessment, as well as the adaptability of these natural materials in both new construction and restoration projects.

Therefore, the Special Issue highlights several robust scientific foundations for the broader use of geomaterials, motivating researchers by offering clear guidance for future research to support the circular economy and protect architectural heritage and advancing performance-based design methods. In the context of civil engineering, environmental and architectural aspects, publications not only broaden current knowledge but also highlight the strategic significance of natural building materials in fostering resilient, resource-efficient, and culturally sensitive built environments.

## References

[1] Sadar Din K.M., Ishak M.S. (2024). Sustainable Building Construction Materials in the United Arab Emirates: A Review. Sustainability.

[2] Din K.M.S., Sayuti Ishak M. (2025). Effects of Project Management Challenges on the Use of Sustainable Construction Materials: Evidence from Construction Industry Practitioners. Civ. Environ. Eng..

[3] Tazmeen T., Mir F.Q. (2024). Sustainability through materials: A review of green options in construction. Results Surf. Interfaces.

[4] Zohbi G.A., Askar S.A., Kumar A., Kumar P., Dahiya M., Rani S. (2024). Sustainable building materials for Eco-friendly construction. AIP Conf. Proc..

[5] Marandi N., Shirzad S. (2025). Sustainable cement and concrete technologies: A review of materials and processes for carbon reduction. Innov. Infrastruct. Solut..

[6] Firoozi A.A., Firoozi A.A., Oyejobi D.O., Avudaiappan S., Flores E.S. (2024). Emerging trends in sustainable building materials: Technological innovations, enhanced performance, and future directions. Results Eng..

[7] Šekularac N., Debljović Ristić N., Mijović D., Cvetković V., Barišić S., Ivanović-Šekularac J. (2019). The Use of Natural Stone as an Authentic Building Material for the Restoration of Historic Buildings in Order to Test Sustainable Refurbishment: Case Study. Sustainability.

[8] Vafaie F., Remøy H., Gruis V. (2023). Adaptive reuse of heritage buildings; a systematic literature review of success factors. Habitat Int..

[9] Valipour A., Manesh M.S., Balali A. (2025). Sustainable material selection for the reconstruction of historical buildings using building information modeling (BIM) in developing countries. Sustain. Cities Soc. Adv..

[10] Manzi S., Mazzotti C., Bignozzi M.C. (2013). Short and long-term behavior of structural concrete with recycled concrete aggregate. Cem. Concr. Compos..

[11] Xing Z., Beaucour A.L., Hebert R., Noumowe A., Ledesert B. (2011). Influence of the nature of aggregates on the behaviour of concrete subjected to elevated temperature. Cem. Concr. Res..

[12] Roufael G., Beaucour A.L., Eslami J., Hoxha D., Noumowé A. (2021). Influence of lightweight aggregates on the physical and mechanical residual properties of concrete subjected to high temperatures. Constr. Build. Mater..

[13] Sadowska-Buraczewska B., Kujawska J. (2021). Comparative Analysis of Physical-Mechanical Properties of Natural and Recycled Aggregate Concretes. Adv. Sci. Technol. Res. J..

[14] Ortiz-Marqués A., Caldevilla P., Goldmann E., Safuta M., Fernández-Raga M., Górski M. (2025). Porosity and Permeability in Construction Materials as Key Parameters for Their Durability and Performance: A Review. Buildings.

[15] Přikryl R. (2021). Geomaterials as construction aggregates: A state-of-the-art. Bull. Eng. Geol. Env..

[16] Majer S., Sołowczuk A., Budziński B. (2024). Long-Term Performance of Natural Stone Cobbles for Paving Raised Junctions: Findings from over a Decade of Use. Sustainability.

[17] Yavuz A.B., Dağ R., Sarı S.A. (2022). Quantification and estimation of the durability of stones used as construction material more precisely by modification of static rock durability index. Constr. Build. Mater..

[18] Lisci C., Sitzia F., Pires V., Aniceto M., Mirão J. (2023). Stone Endurance: A Comparative Analysis of Natural and Artificial Weathering on Stone Longevity. Heritage.

[19] Correa-Jaramillo R., Hernández-Olivares F. (2025). Sustainability in Construction: Geopolymerized Coating Bricks Made with Ceramic Waste. Materials.

[20] Łucarz M., Garbacz-Klempka A., Brzeziński M., Pribulová A., Fedorko P. (2024). Investigation of Spent Moulding Sand Using Thermal Treatment with Regard to the Possibility of Recovering Quartz Matrix. Materials.

[21] Ferraz E., Terroso D., Sequeira M.C., Azevedo M.C., Coroado J., Monteiro C., Rocha F., Gamelas J.A.F. (2024). A Starting Point on Recycling Land and Sea Snail Shell Wastes to Manufacture Quicklime, Milk of Lime, and Hydrated Lime. Materials.

[22] Berger J., Mocciaro A., Cordoba G., Martinefsky C., Irassar E.F., Beuntner N., Scherb S., Thienel K.-C., Tironi A. (2024). Challenges and Performance of Filter Dusts as a Supplementary Cementitious Material. Materials.

[23] Dal Poggetto G., Douwe P., Stroscio A., Kamseu E., Lancellotti I., Elimbi A., Leonelli C. (2024). Dissolution of Volcanic Ash in Alkaline Environment for Cold Consolidation of Inorganic Binders. Materials.

[24] Wang X., Xue Y. (2024). Preparation and Immobilization Mechanism of Red Mud/Steel Slag-Based Geopolymers for Solidifying/Stabilizing Pb-Contaminated Soil. Materials.

[25] Gomah M.E., Li G., Omar A.A., Abdel Latif M.L., Sun C., Xu J. (2024). Thermal-Induced Microstructure Deterioration of Egyptian Granodiorite and Associated Physico-Mechanical Responses. Materials.

[26] Zagaroli A., Kubica J., Galman I., Falkjar K. (2024). Study on the Mechanical Properties of Two General-Purpose Cement–Lime Mortars Prepared Based on Air Lime. Materials.

[27] Valido J.A., Cáceres J.M., Freire-Lista D.M., Sousa L.M.O. (2023). Effect of Salt Mist Ageing on the Physical and Mechanical Properties of Two Ignimbrites from the Canary Islands (Spain). Materials.

[28] Brzyski P., Boris R. (2023). The Influence of Acid Casein on the Selected Properties of Lime–Metakaolin Mortars. Materials.

[29] Strzałkowski P., Köken E., Sousa L. (2023). Guidelines for Natural Stone Products in Connection with European Standards. Materials.

[30] Coviello C.G., La Scala A., Sabbà M.F., Carnimeo L. (2024). On the Cementitious Mixtures Reinforced with Waste Polyethylene Terephthalate. Materials.

